# RECOVERY- Respiratory Support: Respiratory Strategies for patients with suspected or proven COVID-19 respiratory failure; Continuous Positive Airway Pressure, High-flow Nasal Oxygen, and standard care: A structured summary of a study protocol for a randomised controlled trial

**DOI:** 10.1186/s13063-020-04617-3

**Published:** 2020-07-29

**Authors:** Gavin D. Perkins, Keith Couper, Bronwen Connolly, J. Kenneth Baillie, Judy M. Bradley, Paul Dark, Anthony De Soyza, Ellen Gorman, Alasdair Gray, Louisa Hamilton, Nicholas Hart, Chen Ji, Ranjit Lall, Nicola McGowan, Scott Regan, Anita K. Simonds, Emma Skilton, Nigel Stallard, Emily Stimpson, Joyce Yeung, Daniel F. McAuley

**Affiliations:** 1 Warwick Clinical Trials Unit, Warwick Medical School, University of Warwick, Coventry, UK; 2 Critical care unit, University Hospitals Birmingham NHS Foundation Trust, Birmingham, UK; 3 Wellcome Wolfson Institute for Experimental Medicine, School of Medicine, Dentistry and Biomedical Science Queen’s University Belfast, Belfast, UK; 4 The Roslin Institute, University of Edinburgh, Edinburgh, UK; 5 Intensive Care Unit, Royal Infirmary Edinburgh, Edinburgh, UK; 6 Division of Infection, Immunity and Respiratory Medicine, NIHR Manchester Biomedical Research Centre, Faculty of Biology, Medicine and Health, University of Manchester, Manchester, UK; 7 Institute of Cellular Medicine, NIHR Biomedical Research Centre for Ageing, Newcastle University, Newcastle, UK; 8 Department of Respiratory Medicine, Freeman Hospital, Newcastle, UK; 9 Emergency Medicine Research Group of Edinburgh, Royal Infirmary of Edinburgh, Edinburgh, UK; 10 Department of Emergency Medicine, Royal Infirmary of Edinburgh, Edinburgh, UK; 11 Centre for Human and Applied Physiological Sciences, King’s College London, London, UK; 12 Lane Fox Respiratory Service, Guy’s and St Thomas’ NHS Foundation Trust, London, UK; 13 Sleep and Ventilation Unit, Royal Brompton and Harefield NHS Foundation Trust, London, UK; 14 Division of Health Sciences, Warwick Medical School, University of Warwick, Coventry, UK

**Keywords:** COVID-19, Randomised controlled trial, protocol, continuous positive airway pressure, oxygen inhalation therapy, high-flow nasal oxygen

## Abstract

**Objective:**

The trial objective is to determine if Continuous Positive Airway Pressure (CPAP) or High-Flow Nasal Oxygen (HFNO) is clinically effective compared to standard oxygen therapy in patients with confirmed or suspected COVID-19.

**Trial design:**

Adaptive (group-sequential), parallel group, pragmatic, superiority randomised controlled, open-label, multi-centre, effectiveness trial.

**Participants:**

The trial is being conducted across approximately 60 hospitals across England, Wales, Scotland, and Northern Ireland. Inpatients at participating hospitals are eligible to participate if they have respiratory failure with suspected or proven COVID-19, and meet all of the inclusion criteria and none of the exclusion criteria.

Inclusion criteria: 1) Adults ≥ 18 years; 2) Admitted to hospital with suspected or proven COVID-19; 3) Receiving oxygen with fraction of inspired oxygen (FiO_2_) ≥0.4 and peripheral oxygen saturation (SpO_2_) ≤94%; and 4) Plan for escalation to tracheal intubation if needed.

Exclusion criteria: 1) Planned tracheal intubation and mechanical ventilation imminent within 1 hour; 2) Known or clinically apparent pregnancy; 3) Any absolute contraindication to CPAP or HFNO; 4) Decision not to intubate due to ceiling of treatment or withdrawal of treatment anticipated; and 5) Equipment for both CPAP and HFNO not available.

**Intervention and comparator:**

Intervention one: Continuous positive airway pressure delivered by any device. Set-up and therapy titration is not protocolised and is delivered in accordance with clinical discretion.

Intervention two: High-flow nasal oxygen delivered by any device. Set-up and therapy titration is not protocolised and is delivered in accordance with clinical discretion.

Comparator group: Standard care- oxygen delivered by face mask or nasal cannula (excluding the use of continuous positive airway pressure or high-flow nasal oxygen). Set-up and therapy titration is not protocolised and is delivered in accordance with clinical discretion.

Intervention delivery continues up to the point of death, tracheal intubation, or clinical determination that there is no ongoing need (palliation or improvement).

**Main outcomes:**

The primary outcome is a composite outcome comprising tracheal intubation or mortality within 30 days following randomisation.

Secondary outcomes include tracheal intubation rate, time to tracheal intubation, duration of invasive ventilation, mortality rate, time to mortality, length of hospital stay, and length of critical care stay.

**Randomisation:**

Participants are randomised in a 1:1:1 ratio to receive either continuous positive airway pressure, high-flow nasal oxygen or standard care. Due to the challenging environment of study delivery, a specific intervention may not always be available at the hospital site. The study uses two integrated randomisation systems to allow, where required, the site to randomise between all three interventions, between CPAP and standard care, and between HFNO and standard care. System integration ensures maintenance of balance between interventions.

Randomisation is performed using a telephone-based interactive voice response system to maintain allocation concealment. The randomisation sequence was computer-generated using the minimisation method. Participant randomisation is stratified by site, gender (M/F), and age (<50, >=50 years).

**Blinding (masking):**

The nature of the trial interventions precludes blinding of the researcher, patient and clinical team. Primary and secondary outcomes are all objective outcomes, thereby minimising the risk of detection bias.

**Numbers to be randomised (sample size):**

4002 participants (1334 to be randomized to each of the three study arms)

**Trial Status:**

Current protocol: Version 4.0, 29^th^ May 2020. Recruitment began on April 6, 2020 and is anticipated to be complete by April 5, 2021.

The trial has been awarded Urgent Public Health status by the National Institute of Health Research on 13^th^ April 2020.

**Trial registration:**

ISRCTN, ISRCTN16912075. Registered 6^th^ April 2020, http://www.isrctn.com/ISRCTN16912075

**Full protocol:**

The full protocol (version 4.0, 29^th^ May 2020) is attached as an additional file, accessible from the Trials website (Additional file 1). In the interest in expediting dissemination of this material, the familiar formatting has been eliminated; this Letter serves as a summary of the key elements of the full protocol.

The study protocol has been reported in accordance with the Standard Protocol Items: Recommendations for Clinical Interventional Trials (SPIRIT) guidelines (Additional file 2).

## Supplementary information

**Additional file 1.**

**Additional file 2.**

## Data Availability

The investigator team will have full access to the final trial dataset. The trial will be reported in accordance with the Consolidated Standards of Reporting Trials (CONSORT) guidelines. Publications will be published in peer-reviewed open access journals. Requests for data sharing will be reviewed on an individual basis by the chief investigator. The study will comply with the good practice principles for sharing individual participant data from publicly funded clinical trials and data sharing will be undertaken in accordance with the required regulatory requirements.”

