# Supplementary material for: RECOVERY- Respiratory Support: Respiratory Strategies for patients with suspected or proven COVID-19 respiratory failure; Continuous Positive Airway Pressure, High-flow Nasal Oxygen, and standard care: A structured summary of a study protocol for a randomised controlled trial
Source: Trials. 2020 Jul 29;21:687. doi: 10.1186/s13063-020-04617-3 (PMC7388424; doi:10.1186/s13063-020-04617-3)
Supplement: Supplementary file 1 — Additional file 1. [file 13063_2020_4617_MOESM1_ESM.pdf]

PROTOCOL

RECOVERY-RS Respiratory Support: Respiratory Strategies in COVID-19; CPAP, High-flow, and standard care

ISRCTN Number:	ISRCTN16912075
Sponsor:	University of Warwick
Funding Body:	NIHR
Ethics Approval date:	3rd April 2020
Version Number:	4.0
Date:	29th May 2020

This protocol has regard for current HRA guidance and content

Protocol Amendments:

Amendment No.	Date of Amendment	Date of Approval
1	21st April 2020	21st April 2020
2	24th April 2020	24th April 2020
3	29th May 2020	1st June 2020

CONTACT NAMES AND NUMBERS

Role	Name, address, telephone
**Sponsor:**	Jane Prewett<br>Research and Impact Services, University House<br>University of Warwick<br>Gibbet Hill Road<br>Coventry, CV4 7AL<br>Tel: 02476 522746<br>Email: Jane.prewett@warwick.ac.uk
**Chief Investigator:**	Professor Gavin Perkins<br>Warwick Clinical Trials Unit (WCTU)<br>The University of Warwick<br>Gibbet Hill Road<br>Coventry, CV4 7AL<br>Tel: 02476 750479<br>Email: g.d.perkins@warwick.ac.uk
**Co-Chief Investigator:**	Professor Danny McAuley<br>Wellcome Wolfson Institute for Experimental Medicine<br>Queen's University Belfast<br>97 Lisburn Road<br>Belfast, BT9 7BL<br>Tel: 028 9097 6385<br>Email: d.f.mcauley@qub.ac.uk
**Trial Manager**	Trial Manager<br>Warwick Clinical Trials Unit (WCTU)<br>The University of Warwick<br>Gibbet Hill Road<br>Coventry, CV4 7AL<br>Email: recovery-rs@warwick.ac.uk

Role	Name, address, telephone
**Co-investigators:**	Dr Kenny Baillie<br>Roslin Institute, University of Edinburgh<br>Easter Bush<br>Midlothian, EH25 9RG<br>Tel: 0131 651 9100<br>Email: kennethbaillie@gmail.com<br><br>Professor Judy Bradley<br>Wellcome Trust-Wolfson Northern Ireland Clinical Research Facility<br>U Floor, Belfast City Hospital<br>Lisburn Road<br>Belfast, BT9 7AB<br>Tel: 028 950 47973<br>Email: judy.bradley@qub.ac.uk<br><br>Dr Bronwen Connolly<br>Wellcome-Wolfson Institute for Experimental Medicine<br>Queen's University Belfast<br>97 Lisburn Road<br>Belfast, BT9 7BL<br>Tel: 028 9097 6047<br>Email: b.connolly@qub.ac.uk<br><br>Dr Keith Couper<br>Warwick Clinical Trials Unit (WCTU)<br>The University of Warwick<br>Gibbet Hill Road<br>Coventry, CV4 7AL<br>Tel: 02476 151179<br>Email: k.couper@warwick.ac.uk<br><br>Professor Anthony de-Soyza<br>Cardiothoracic Directorate<br>Freeman Hospital<br>The Newcastle Upon Tyne Hospitals NHS Foundation Trust<br>High Heaton<br>Newcastle upon Tyne, NE7 7DN<br>Tel: 0191 213 7468<br>Email: anthony.de-soyza@newcastle.ac.uk

Role	Name, address, telephone
	Dr Ellen Gorman<br>Wellcome Wolfson Institute for Experimental Medicine<br>Queen's University Belfast<br>97 Lisburn Road<br>Belfast, BT9 7BL<br>Tel: 028 9097 1643<br>Email: egorman03@qub.ac.uk<br><br>Professor Paul Dark<br>Division of Infection, Immunity, and Respiratory Medicine<br>NIHR Manchester Biomedical Research Centre<br>Faculty of Biology, Medicine and Health<br>University of Manchester<br>Email: paul.m.dark@manchester.ac.uk<br><br>Professor Alasdair Gray<br>Professor of Emergency Medicine<br>Edinburgh Royal Infirmary<br>51 Little France Crescent<br>Edinburgh, EH16 4SA<br>Email: alasdair.gray@luht.scot.nhs.uk<br><br>Professor Nicholas Hart<br>Lane Fox Respiratory Service, Guy's and St.Thomas' NHS Foundation Trust<br>St.Thomas' Hospital, Westminster Bridge Road<br>London, SE1 7EH<br>Tel: 020 7188 6115<br>Email: nicholas.hart@gstt.nhs.uk<br><br>Professor Ranjit Lall<br>Warwick Clinical Trials Unit (WCTU)<br>The University of Warwick<br>Gibbet Hill Road<br>Coventry, CV4 7AL<br>Tel: 02476 574659<br>Email: r.lall@warwick.ac.uk

Role	Name, address, telephone
	Professor Anita Simonds<br>Department of Respiratory Medicine, Royal Brompton Hospital<br>Royal Brompton & Harefield NHS Foundation Trust<br>Sydney Street<br>London, SW3 6NP<br>Tel: 020 7352 8121<br>Email: A.Simonds@rbht.nhs.uk<br><br>Professor Nigel Stallard<br>Department of Statistics and Epidemiology<br>Warwick Medical School<br>University of Warwick<br>Coventry, CV4 7AL<br>Tel: 02476 575130<br>Email: N.Stallard@warwick.ac.uk<br><br>Dr Joyce Yeung<br>Warwick Clinical Trials Unit (WCTU)<br>The University of Warwick<br>Gibbet Hill Road<br>Coventry, CV4 7AL<br>Tel: 02476 574880<br>Email: J.Yeung.4@warwick.ac.uk
**Statistician:**	Dr Chen Ji<br>Warwick Clinical Trials Unit (WCTU)<br>The University of Warwick<br>Gibbet Hill Road<br>Coventry, CV4 7AL<br>Tel: 02476 574659<br>Email: C.Ji.3@warwick.ac.uk
**Trial Steering Committee:**	A list of members and contact details can be found in the Trial Master File
**Data Monitoring Committee:**	A list of members and contact details can be found in the Trial Master File
For general queries and supply of trial materials please contact the coordinating centre:<br>Warwick Clinical Trials Unit (WCTU)<br>The University of Warwick<br>Gibbet Hill Road<br>Coventry<br>CV4 7AL<br>Email: recovery-rs@warwick.ac.uk	

TABLE OF CONTENTS PAGE

TABLE OF CONTENTS ... 7
TRIAL SUMMARY ... 10
LIST OF ABBREVIATIONS/GLOSSARY ... 11
1. BACKGROUND ... 12
1.1 Epidemiology and burden of the condition ... 12
1.2 Existing knowledge ... 13
1.3 Hypothesis ... 15
1.4 Need for a trial ... 16
1.5 Ethical considerations ... 16
1.6 CONSORT ... 17
2. TRIAL DESIGN ... 17
2.1 Study setting ... 17
2.2 Trial summary and flow diagram ... 18
2.3 Aims and objectives ... 20
2.3.1 Primary objective ... 20
2.3.2 Secondary objective ... 20
2.4 Outcome measures ... 20
2.4.1 Efficacy ... 20
2.4.2 Safety ... 21
2.5 Eligibility criteria ... 21
2.5.1 Inclusion criteria ... 21
2.5.2 Exclusion criteria ... 21
2.6 Participant identification / Screening ... 21
2.7 Site Staff Training ... 22
2.8 Informed consent ... 22
2.8.1 Consent following initial enrolment (for England, Wales and Northern Ireland only) ... 26
2.9 Randomisation ... 27
2.9.1 Randomisation ... 27
2.9.2 Post-randomisation withdrawals, exclusions and Moves out of Region ... 28
2.10 Trial treatments / intervention ... 28
2.10.1 Continuous Positive Airway Pressure arm ... 28
2.10.2 High Flow Nasal Oxygen ... 28
2.10.3 Standard care ... 28
2.11 Blinding and contamination ... 28
2.11.1 Methods for ensuring blinding ... 28

2.11.2 Contamination 29
2.12 End of trial 29
3 METHODS AND ASSESSMENTS 29
3.1 Schedule of delivery of intervention and data collection 29
4 ADVERSE EVENT MANAGEMENT 30
4.1 Definitions 30
4.1.1 Adverse Events (AE) 30
4.1.2 Serious Adverse Events (SAEs) 30
4.1.3 Assessing and Reporting AEs, SAEs and related SAEs 31
4.1.4 SAEs Exempt from reporting 32
4.2 Responsibilities 32
4.3 Notification of deaths 33
4.4 Reporting urgent safety measures 34
5 DATA MANAGEMENT 34
5.1 Data collection and management 34
5.2 Database 34
5.3 Data storage 35
5.4 Data access and quality assurance 35
5.5 Archiving 35
6 STATISTICAL ANALYSIS 35
6.1 Power and sample size 35
6.2 Statistical analysis of efficacy and harms 36
6.2.4 Statistics and data analysis 36
6.2.5 Planned recruitment rate 37
6.3 Health Economic Evaluation 37
7 TRIAL ORGANISATION AND OVERSIGHT 38
7.1 Sponsor and governance arrangements 38
7.2 Ethical approval 38
7.3 Trial Registration 38
7.4 Notification of serious breaches to GCP and/or trial protocol 38
7.5 Indemnity 39
7.6 Trial timetable and milestones 39
7.7 Administration 40
7.8 Trial Management Group (TMG) 40
7.9 Trial Steering Committee (TSC) 40
7.10 Data Monitoring Committee (DMC) 41

7.11 Essential Documentation 41
7.12 Financial Support 41
8 MONITORING, AUDIT AND INSPECTION 42
9 CO-ENROLMENT 42
10 PATIENT AND PUBLIC INVOLVEMENT (PPI) 42
11 CONFIDENTIALITY 43
12 DISSEMINATION AND PUBLICATION 43
13 RESILIENCE 44
14 REFERENCES 45

LIST OF TABLES PAGE

Table 1.	Mental Capacity Act Emergency Consent framework	22-23
Table 2.	Trial assessments	27
Table 3.	Relationship of SAEs to trial intervention	29
Table 4.	Critical values expressed as one-sided p-values for a test with four equally spaced looks	34

LIST OF FIGURES PAGE

Figure 1. Trial flow diagram 18
Figure 2. Consent processes in England, Wales and Northern Ireland........24
Figure 3. Consent processes in Scotland........25

TRIAL SUMMARY

Trial Title	RECOVERY-RS Respiratory Support: Respiratory Strategies in COVID-19; CPAP, High-flow, and standard care	
Internal ref. number (or short title)	RECOVERY-Respiratory Support	
Clinical Phase	Phase III	
Trial Design	Adaptive (group-sequential), pragmatic, randomised controlled, open-label, multi-centre, effectiveness trial	
Trial Participants	Adult, critically ill patients, with suspected or confirmed, COVID-19	
Planned sample size	4002	
Treatment Duration	30 days	
Follow-up Duration	30 days or hospital discharge, whichever comes latest	
Planned Trial Period	18 months	
	Objectives	Outcome Measures
Primary	To determine if CPAP or HFNO is clinically effective compared to standard care (excluding CPAP or HFNO)	Intubation and mortality
Secondary	To assess in-hospital patient data in terms of their stay	Intubation rates; time to intubation; time to death (mortality); mortality in critical care (Level 2/3); mortality in hospital stay; mortality at 30 days; admission to ICU; length of stay in critical care (Level 2/3); length of stay in hospital

LIST OF ABBREVIATIONS/GLOSSARY

Abbreviation	Explanation
AE	Adverse event
ARF	Acute respiratory failure
BiPAP	Bilevel positive airway pressure (BiPAP)
CI	Chief Investigator
CONSORT	*Con*solidated *S*tandards *o*f *R*eporting *T*rials
COVID-19	Coronavirus Disease 2019
CPAP	Continuous positive airway pressure
CRF	Case Report Form
CTU	Clinical Trials Unit
DMC	Data Monitoring Committee
GCP	Good Clinical Practice
HFNO	High flow nasal oxygen
ICF	Informed Consent Form
ICNARC	Intensive Care National Audit and Research Centre
ICU	Intensive Care Unit
IRAS	Integrated Research Application System
ISRCTN	International Standard Randomised Controlled Trial Number
MRC	Medical Research Council
NIV	Non-invasive ventilation
NNT	Number needed to treat
PI	Principal Investigator
PPI	Patient & Public Involvement
RCT	Randomised Controlled Trial
REC	Research Ethics Committee
R&D	Research and Development
SAE	Serious Adverse Event
SARS-CoV-2	Severe acute respiratory syndrome coronavirus 2
SOP	Standard Operating Procedure
TSC	Trial Steering Committee
WCTU	Warwick Clinical Trials Unit

1. BACKGROUND

1.1 Epidemiology and burden of the condition

Severe acute respiratory syndrome coronavirus 2 (SARS-CoV-2) emerged at the end of 2019 as a novel coronavirus, resulting in a current global pandemic of respiratory illness, Coronavirus Disease 2019 (COVID-19). Using very recent World Health Organization (WHO) data on the cumulative number of deaths up to 1 March 2020, mortality rates of 5·6% (95% CI: 5·4%–5·8%) for China and 15·2% (12·5%–17·9%) outside of China have been reported[1]. Indeed, as of 29 March 2020, there have been 575,444 confirmed cases, with 26,654 confirmed deaths, across 202 countries (available at https://www.who.int/emergencies/diseases/novel-coronavirus-2019). National statistics detailed by the Centre of Evidence Based Medicine, University of Oxford, report all-cause mortality by respiratory disease, across England and Wales, during 3 January 2020 to 13 March (i.e. during the COVID-19 outbreak) have averaged approximately 15%[2], albeit peak pandemic phase has not yet occurred in the UK. A recent (27 March 2020) report from the Intensive Care National Audit and Research Centre (ICNARC), reported 846 admissions to critical care units in England, Wales, and Northern Ireland with confirmed COVID-19; where 24 hour outcome data were available in 775 patients, 79 had died, 86 were discharged alive, and 609 patients remained in critical care[3].

COVID-19 has a wide spectrum of clinical severity, ranging from asymptomatic to critically ill, and ultimately death. A common and prominent complication of advanced COVID-19 is acute hypoxemic respiratory insufficiency requiring oxygen and ventilation therapies. Often deterioration may be rapid. A recent report showed that 14% of patients developed dyspnea, tachypnea with a respiratory rate greater than or equal to 30 per minute, desaturation with peripheral oxygen saturation (Sp_{O_2}) less than or equal to 93%, poor oxygenation with a ratio of partial pressure of arterial oxygen (Pa_{O_2}) to fraction of inspired oxygen (F_{IO_2}) less than 300 mmHg, or lung infiltrates greater than 50%, within 48 h[4].

Ventilatory support is the cornerstone of management for COVID-19 patients presenting with respiratory impairment. Reports from China, the source of the pandemic, suggest a prevalence of hypoxic respiratory failure in patients with COVID-19 of 19%[5], although true global figures are unclear. That said, there is evidence that patients have received as standard care management in the form of supplemental oxygen therapy, high-flow nasal oxygen (HFNO), continuous positive airways pressure (CPAP), non-invasive positive pressure ventilation (NIV), and invasive mechanical ventilation[6]. Standard care treatment for patients requiring ventilatory support may involve more than one method and a cross over between non-invasive and invasive devices.

Already the potential impact of the respiratory symptomology associated with COVID-19 on UK critical care units can be appreciated; nearly 80% of the patients in the aforementioned ICNARC report required invasive mechanical ventilation, compared with just over 40% in a non-COVID-19 cohort of viral pneumonia between 2017-2019[3]. However the ability to deliver this treatment at this scale (and potentially greater) across UK critical care settings places huge pressures on existing services with finite resources. This scenario of excess demand on limited ventilator availability and critical care bed capacity is currently being witnessed worldwide, in particular in neighbouring European countries.

Determining the most effective form of ventilatory support for patients with COVID-19 is therefore paramount to ensure optimum therapy is delivered to patients, and to protect UK vital critical care resources and infrastructure. Findings from this trial will also have the potential to inform global ventilation practice for patients with COVID-19.

1.2 Existing knowledge

High flow nasal oxygen

There is currently no direct evidence to support the use of HFNO in patients with COVD-19[6]; instead evidence around the possible application of this therapy in COVID-19 is derived from the acute respiratory failure (ARF) population where it appears inconsistent overall. The most recent relevant Cochrane systematic review and meta-analysis from 2017 demonstrated that the evidence to support the use of HFNO for acute respiratory failure was insufficient to conclude, whether HFNO provided safe and effective respiratory support to these patients[7]. The majority of trials (10 out of 11) included in this review compared HFNC versus low-flow oxygen. This review was unable to demonstrate whether HFNO was more effective or safe than other oxygen delivery devices. For the primary outcomes of treatment failure, defined as need for non-invasive or invasive ventilation (1066 participants; six studies) and mortality (755 participants; three studies), investigators found no differences between HFNO and low-flow oxygen therapies (risk ratio (RR), Mantel-Haenszel (MH), random-effects 0.79, 95% confidence interval (CI) 0.49 to 1.27; and RR, MH, random-effects 0.63, 95% CI 0.38 to 1.06, respectively). There was no evidence that HFNO reduced the rate of treatment failure or risk of death compared with low-flow oxygen devices, and also no evidence of any advantage for HFNO in terms of adverse event rates, ICU length of stay, or duration of respiratory support. In this review the data comparing HFNO to CPAP or Bilevel positive airway pressure(BiPAP) was very limited.

In another recent systematic review and meta-analysis comparing HFNO with usual care, there was no difference in mortality (HFNO, 60/1,006 [6%] vs usual care, 90/1,106 [8.1%]) (n = 2,112; p = 0.29;

I2, 25%; fixed effect model: odds ratio, 0.83; 95% CI, 0.58–1.17) or rate of intubation (HFNC, 119/1,207 [9.9%] vs usual care, 204/1,300 [15.7%]) (n = 2,507; p = 0.08; I2, 53%; random effect model: odds ratio, 0.63; 95% CI, 0.37–1.06)[8]. A qualitative analysis of 13 studies on tolerability and comfort suggested that HFNO was associated with improved patient comfort and dyspnea scores[8]. In contrast, other recent systematic reviews and meta-analyses have demonstrated a benefit of HFNO in reducing intubation compared with conventional oxygen (RR 0.85, 95% CI 0.74 to 0.99), but similarly no effect on risk of death or ICU length of stay[9-11]. Reducing the need for intubation is a clinically meaningful outcome, with additional important benefits for healthcare resource utilisation.

Current Surviving Sepsis Campaign (SSC) COVID-19 guidelines make a weak recommendation, based on low quality evidence, for using HFNO over conventional oxygen therapy[6]. A similar strength of recommendation is made for using HFNO over NIV[6], based on contrasting evidence around the effectiveness for HFNO to reduce mortality, need for intubation, and ICU length of stay[9 12] and potential for greater patient tolerability for HFNO [12]. Guidelines from the Australia and New Zealand Intensive Care Society (ANZICS) similarly recommend HFNO for patients presenting with hypoxia[13]. Both sets of guidelines do however, advise close monitoring of patients receiving either HFNO in a setting of sufficient acuity to enable controlled intubation in the event of deterioration given the high risk of failure[6 13].

Continuous positive airway pressure

There are currently no data available on the use of CPAP directly in patients with COVID-19. A number of trials have explored the effectiveness of CPAP in ARF. These trials provide some evidence that CPAP has a role in improving important outcomes such as progression to intubation, length of stay in ICU and/or hospital. In a 2015 Cochrane systematic review and meta-analysis, Faria *et al*[14] compared the effectiveness of NIV i.e. continuous positive airway pressure (CPAP) or bi-level NIV, in reducing mortality and the rate of tracheal intubation in adults with acute respiratory failure after upper abdominal surgery, compared to standard therapy (oxygen therapy). Two trials involving 269 participants were included and CPAP or bi-level NIV reduced the rate of tracheal intubation (risk ratio (RR) 0.25; 95% confidence interval (CI) 0.08 to 0.83 with a number needed to treat (NNT) for additional benefit of 11. There was very low quality evidence that the intervention may also reduce ICU length of stay (mean difference (MD) -1.84 days; 95% CI -3.53 to -0.15) however there were no differences for mortality (low quality evidence) and hospital length of stay.

Bakke *et al*[15] explored the effectiveness of CPAP and NIV versus standard medical treatment in prehospital treatment of patients with acute respiratory failure in terms of mortality, hospital

length of stay, intensive care unit length of stay or intubation rate. Four studies in this review were high quality studies comparing CPAP to standard care, with only 1 trial showing a reduction in both mortality and intubation rate. The remaining 3 high quality studies showed no significant differences in any of the outcomes of interest albeit showing a trend toward lower intubation rate with supplementary CPAP. Four studies in this review compared NIV to standard medical treatment, and found no difference in mortality or intubation rate, and two studies demonstrated a reduction in ICU stay. Williams *et al*[16] included studies comparing CPAP (and usual care) with no CPAP for ARF in the prehospital setting; a meta-analysis included 5 studies (3 RCTs, 1 non-randomised comparative study, and 1 retrospective comparative study) totalling 1002 patients and showed pooled estimates demonstrated significantly fewer intubations ([OR] 0.31; 95% confidence interval [CI] 0.19-0.51) and lower mortality (OR 0.41; 95% CI 0.19-0.87) in the CPAP group.

Current international guidelines do not address the use of CPAP in COVID-19 patients, focusing instead on HFNO, NIV, and invasive mechanical ventilation once patients are intubated. In contrast, national guidance from NHS England state that CPAP is the preferred form of non-invasive ventilator support; HFNO is not advocated due to lack of efficacy[17].

Non-invasive ventilation

There is no direct evidence to support the use of NIV in patients with hypoxic respiratory failure, with some reports of increased risk to healthcare workers of exposure to infection[6]. Previous meta-analyses where reductions in intubation and mortality have been demonstrated with NIV have been in patient populations less relevant to COVID-19[6]. Failure rates are generally high with use of NIV outside of cardiogenic pulmonary oedema. Should other pathophysiology be present e.g. acute hypercapnic respiratory failure, NIV may be considered[13 17-19]. Surviving Sepsis Campaign guidelines recommend HFNO over NIV[6], based on contrasting evidence around the effectiveness for HFNO to reduce mortality, need for intubation, and ICU length of stay[9 12] and potential for greater patient tolerability for HFNO[12]. In patients with COVID-19, where HFNO is not available and there is no urgent need for intubation, a trial of NIV with close monitoring and short-interval assessment for deterioration may be considered (weak recommendation, very low quality evidence)[6].

1.3 Hypothesis

In adult patients, with suspected or confirmed COVID-19, CPAP or HFNO are more effective than standard care for reducing the rate of intubation and / or mortality.

We will aim to recruit a maximum of 4002 patients to explore this hypothesis. However, as this is an adaptive trial (group-sequential) in design, we will review the data at each stage using formal

interim analyses and continue with all arms or drop an intervention arm if required. The trial will therefore stop early and not require this total sample size if an intervention appears to be effective at an interim analysis.

1.4 Need for a trial

This review of the literature highlights that whilst there is some evidence to support current ventilation options for patients with COVID-19, there is little evidence to guide the relative efficacy of these interventions especially HFNO versus CPAP versus standard care oxygen therapy prior to intubation. Importantly, evidence is derived from the general ARF population; there are no COVID-19-specific data. Evidence on the relative effectiveness of these interventions is paramount to ensuring that critically ill patients with COVID-19 are optimally managed in terms of oxygen requirements and that progression to intubation is minimised. There is clear clinical uncertainty as evidenced in the contrasting recommendations amongst existing sets of recommendations[6 13 17].

In selecting the interventions for investigation in this trial, we have elected to compare the effects of HFNO and CPAP against standard care. We have elected not to examine NIV due to the uncertainty around its applicability to the COVID-19 population and the clinical nature of respiratory impairment in these patients. This trial will provide important data to clarify current national recommendations on the preferred mode of non-invasive respiratory support[17].

We have also reviewed the experiences to date from other countries and relevant literature to support treatment options in patients admitted with suspected or confirmed COVID-19 and developing hypoxia. Yang *et al*[20] have recently published the first series of 52 patients from the China COVID-19 crisis. They highlight that HFNO and mechanical ventilation (either non-invasive or invasive) was the main supportive treatment for these critically ill patients. Many practical drivers have been highlighted that influence the use of these options. It is a deep concern in some areas that the demand for oxygen therapy will outweigh the ability to provide it to patients. For those interventions that require equipment there is huge dearth of equipment available and for some interventions (such as NIV) there is substantial specialist training required to deliver the intervention optimally. This is balanced against the absolute need to reduce the number of patients requiring intubation given the imbalance between ventilator provision and likely demand.

1.5 Ethical considerations

The trial will recruit patients in UK-based hospitals and centres to provide care to COVID-19 patients in England, Wales, Scotland and Northern Ireland, many of whom will lack capacity to consent due

to the nature of the underlying disease process. It is likely that patients eligible for the trial will lack capacity, primarily due to acute confusion secondary to hypoxia and infection.

Patients will be assessed for capacity in the first instance. Those with capacity will be provided with information about the trial and asked to give verbal consent prior to enrolment. Given the urgent need to initiate treatment and the context in which the trial will operate (e.g. restricted visiting and infection control risks) , if the patient does not have capacity, it is not reasonably practicable to consult a relative / guardian / consultee or a medical practitioner independent of the research team. In England, Wales and Northern Ireland, legislation permits the Research Ethics Committee to authorise enrolment where action needs to be taken as a matter of urgency and to subsequently seek consent. This approach is guided by a published framework, developed in collaboration with the National Research Ethics Service for use in research in an emergency setting[21]. For sites in Scotland, if a patient does not have capacity, the legislation does not allow enrolment without consent from the patient or guardian.

The trial will be conducted in full conformance with the principles of the Declaration of Helsinki and to Good Clinical Practice (GCP) guidelines. It will also comply with all applicable UK legislation, the Emergency framework for deferred consent (for England, Wales and Northern Ireland), developed by Davies *et al*[21] in conjunction with the National Research Ethics Service, and Warwick Standard Operating Procedures (SOPs). Adults without capacity will not be recruited from Scotland. All data will be stored securely and held in accordance with the Data Protection Act 2018.

1.6 CONSORT

The trial will be reported in line with the CONSORT (*Con*solidated *S*tandards *o*f *R*eporting *T*rials) statement (Lancet 2001, 357: 1191-1194, www.consort-statement.org).

2. TRIAL DESIGN

2.1 Study setting

The main trial will take place in NHS hospitals (in England, Wales, Scotland and Northern Ireland) or purpose built-hospitals with wards and critical care areas that have access to a population of patients with confirmed or suspected COVID-19, and where consultants and staff managing the patient have clinical equipoise for the use of trial interventions and agree to maintain trial allocation in randomised patients.

2.2 Trial summary and flow diagram

The trial will be an adaptive (group-sequential), pragmatic, randomised controlled, open-label, multi-centre, effectiveness trial to determine if the use of CPAP or HFNO reduces intubation or death (mortality), within 30 days. We will test each of the intervention against standard care, and if required, drop one of the interventions if there is evidence of harm or ineffectiveness.

Patients with suspected or confirmed COVID-19 will be eligible for randomisation when $FiO_2 \geq 0.4$ and $SpO_2 \leq 94\%$, in a 1:1:1 ration to receive either CPAP, HFNO, or standard care (excluding either of the two treatments). Data will be recorded by participating ICUs until hospital discharge, and all surviving patients will be followed up to 30 days.

Figure 1. **Trial flow diagram**

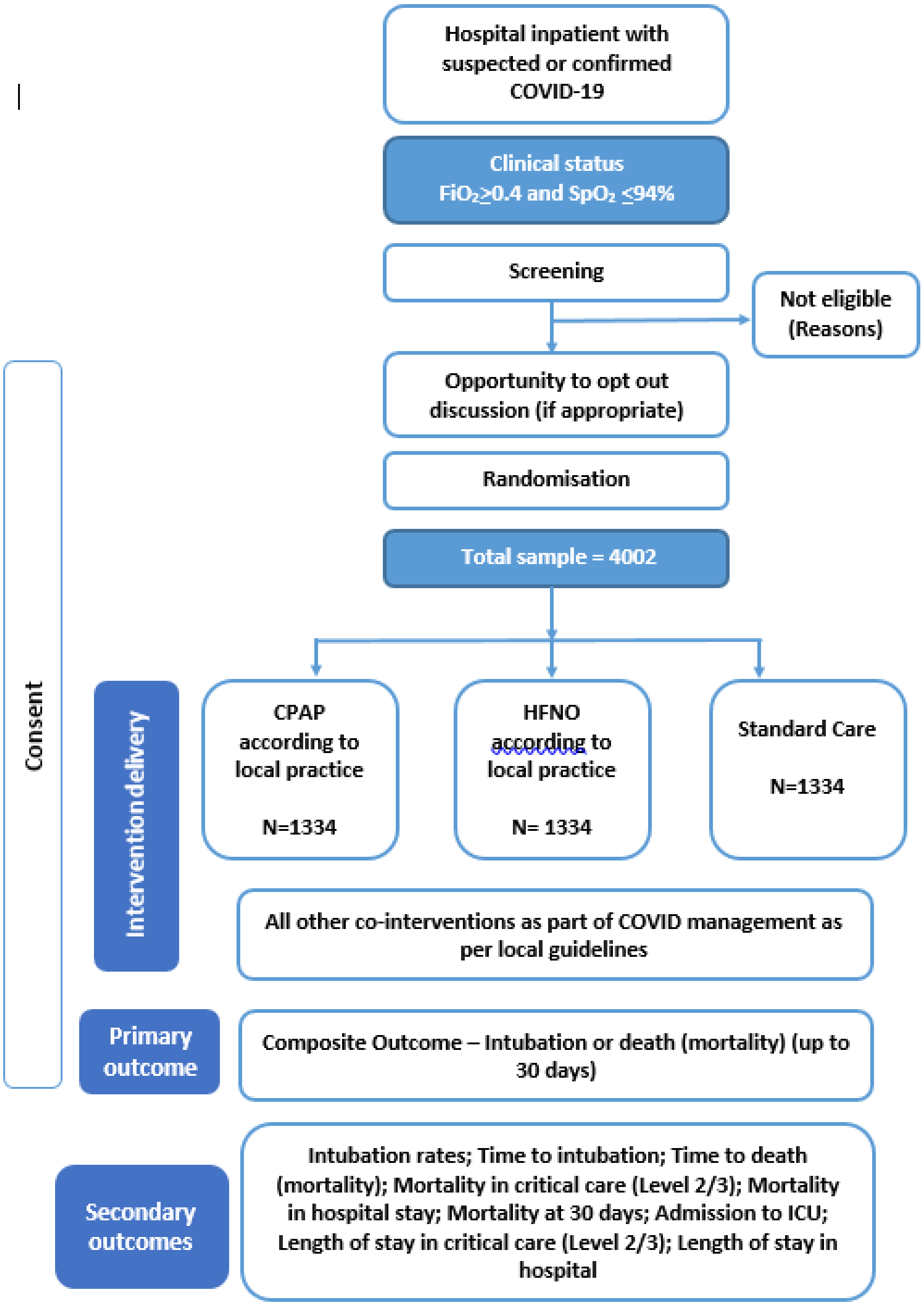

2.3 Aims and objectives

2.3.1 Primary objective

The primary objective of this trial is to determine if CPAP or HFNO is clinically effective compared to standard care in relation to intubation and mortality in patients with confirmed or suspected COVID-19.

2.3.2 Secondary objective

The secondary objectives for this study are to assess in-hospital patient data in terms of their stay.

2.4 Outcome measures

2.4.1 Efficacy

- *Primary*

  The primary outcome is a composite outcome comprising intubation or mortality within 30 days. If necessary, patients will be followed up to ascertain all-cause mortality rates at 30 days, using NHS Digital, the Intensive Care National Audit and Research Centre (ICNARC) and equivalent NHS data linkage in the devolved nations. Intubation will be obtained from hospital data.

- *Secondary outcomes*

  Secondary outcomes relating to effectiveness include:

  - Intubation rates
  - Time to intubation
  - Duration of invasive ventilation
  - Time to death (mortality)
  - Mortality in critical care (level 2/3)
  - Mortality in hospital stay
  - Mortality at 30 days
  - Admission to ICU
  - Length of stay in critical care (level 2/3)
  - Length of stay in hospital

  All secondary outcomes will be obtained from hospital records and data linkage where appropriate.

2.4.2 Safety

We will collect data on:

- Adverse events
- Serious adverse events

2.5 Eligibility criteria

The eligibility criteria are designed to include those who reflect the general population of patients with confirmed or suspected COVID-19 and who may benefit from the therapeutic interventions. Patients are eligible to be included in the trial if they meet the following criteria:

2.5.1 Inclusion criteria

1. Adults ≥ 18 years
2. Hospital inpatient with suspected or proven COVID-19
3. FiO_2 $\geq$0.4 and SpO_2 $\leq$94%
4. Plan for escalation to intubation if needed

2.5.2 Exclusion criteria

1. Planned intubation and mechanical ventilation imminent within 1 hour
2. Known or clinically apparent pregnancy
3. Any absolute contraindication to CPAP or HFNO
4. Decision not to intubate due to ceiling of treatment or withdrawal of treatment anticipated
5. Equipment for both CPAP and HFNO not available

2.6 Participant identification / Screening

During the study recruitment period, hospital research teams will, on a regular basis, attend or contact hospital clinical areas caring for patients with known or suspected COVID-19. Teams will liaise with clinical staff to identify individuals with acute hypoxaemic respiratory failure that may be eligible for enrolment. Based on this referral, a member of the team delivering the trial at the hospital (e.g. doctor, nurse, research practitioner) with appropriate knowledge will formally assess eligibility of the patient against trial inclusion and exclusion criteria. No additional tests or investigations will be required for assessing eligibility.

2.7 Site Staff Training

We will be using the emergency research consent process. The PI will retain overall responsibility for conduct of the trial at their site.

2.8 Informed consent

<u>England, Wales and Northern Ireland</u>

Patients will be assessed for capacity. Patients with capacity will be provided with verbal information about the trial and given a copy of the written participant information sheet. They will be given adequate time to review the information sheet and ask questions. Due to ongoing concerns regarding risk of infection transmission, verbal consent only will be sought and documented on a study form and in the patient hospital records. Where a patient objects at this point they will not be entered into the study.

For patients who lack capacity, it is not practical to consult a personal consultee or independent registered medical practitioner prior to enrolment without placing the potential participant at risk of harm from delaying treatment. These processes will also not be feasible or safe to undertake in order to minimise risk of viral transmission to those involved. Significant limitations have been placed on the visitation of COVID-19 patients and therefore a personal consultee is unlikely to immediately be available, and to attempt contact via telephone will delay treatment. Pressures on clinical services will make it impractical to identify an appropriate independent medical practitioner. In accordance with the legislation in England, Wales and Northern Ireland, as action needs to be taken as a matter of urgency, the Research Ethics Committee has authorised enrolment prior to obtaining informed consent. Once the urgency of the situation has passed, consent to continue will be sought from the patient (if capacity returns) or consultee. This approach is guided by a published framework, developed in collaboration with the National Research Ethics Service for use in research in an emergency setting[21]. For sites in Scotland, if a patient does not have capacity, the legislation does not allow enrolment without consent from the patient or guardian.

It is also important to highlight that all of the interventions proposed in the trial (CPAP, HFNO, and standard care) are existing standards of care currently employed in clinical practice; we are not investigating any experimental interventions in this trial.

Further details of the rationale for our approach are detailed in Table 2 and can be seen in figure 2, and our steps for obtaining consent following initial enrolment are described in Section 2.8.1.

Table 1: Mental Capacity Act Emergency Consent framework (for England, Wales and Northern Ireland only)

1. Is this research needed?	
	There is an urgent need to determine the optimum strategy to manage COVID-19 patients with worsening respiratory failure. This proposal has been commissioned to answer a research question that is considered to be of high priority by the UK clinical community.
2. Is there uncertainty about treatment?	
	There are perceived benefits to each study intervention, and each is in widespread use across the NHS. However, there is ongoing uncertainty as to the optimal approach
3. Is there a need to recruit subjects who lack capacity?	
	Eligible participants will be critically unwell and are likely to lack capacity as part of their underlying condition (e.g. acute hypoxia, sepsis). These individuals are an important COVID-19 group that are potentially at greater risk of deterioration.
4. In the context of the research is consent or consultation feasible?	
	We have carefully considered the feasibility of seeking agreement from a personal or professional consultee in the context of this research.<br>- Personal consultee- throughout the NHS, significant limitations have been placed on the visitation of COVID-19 patients. On this basis, a personal consultee will not be immediately physical available for consultation. Waiting for a personal consultee to attend the hospital would place the consultee at risk of infection and cause treatment delays.<br>- Professional consultee- due to COVID-19, there will be extreme pressure on UK health services. In this context, we consider it impractical for hospital research teams to identify an appropriate medical practitioner to act as a professional consultee without delaying treatment.
5) Does treatment need to be given quickly?<br>6) Might delay change the effect of treatment or the results?	
	It is essential that treatment (as per the study arms) is initiated as soon as possible. Delays in treatment initiation may place the patient at risk, and increase the likelihood of the need for tracheal intubation and increase the risk of death. Delays may therefore make study interventions appear less effective by reducing any observed treatment effect.
7. Will procedures accommodate variations in capacity?	
	Patients will be assessed for capacity in the first instance and provided with the patient information sheet detailing the trial, the patient will be given adequate time to consider the trial before providing verbal consent.

8. Would the legal representative/consultee be likely to have capacity?	
	A consultee may have capacity, but consultation is impractical due to the urgent need to commence treatment and research context.
9. Is it practical to consult a professional legal representative unconnected to the research?	
	As per question four, the research context makes it impractical to consult a professional consultee.
10. What should the patient, consultee or legal representative be asked later?	
	We will subsequently ask for patient consent or consultee agreement for ongoing delivery of the intervention (if applicable) and ongoing data collection.

Figure 2. Consent processes for England, Wales and Northern Ireland

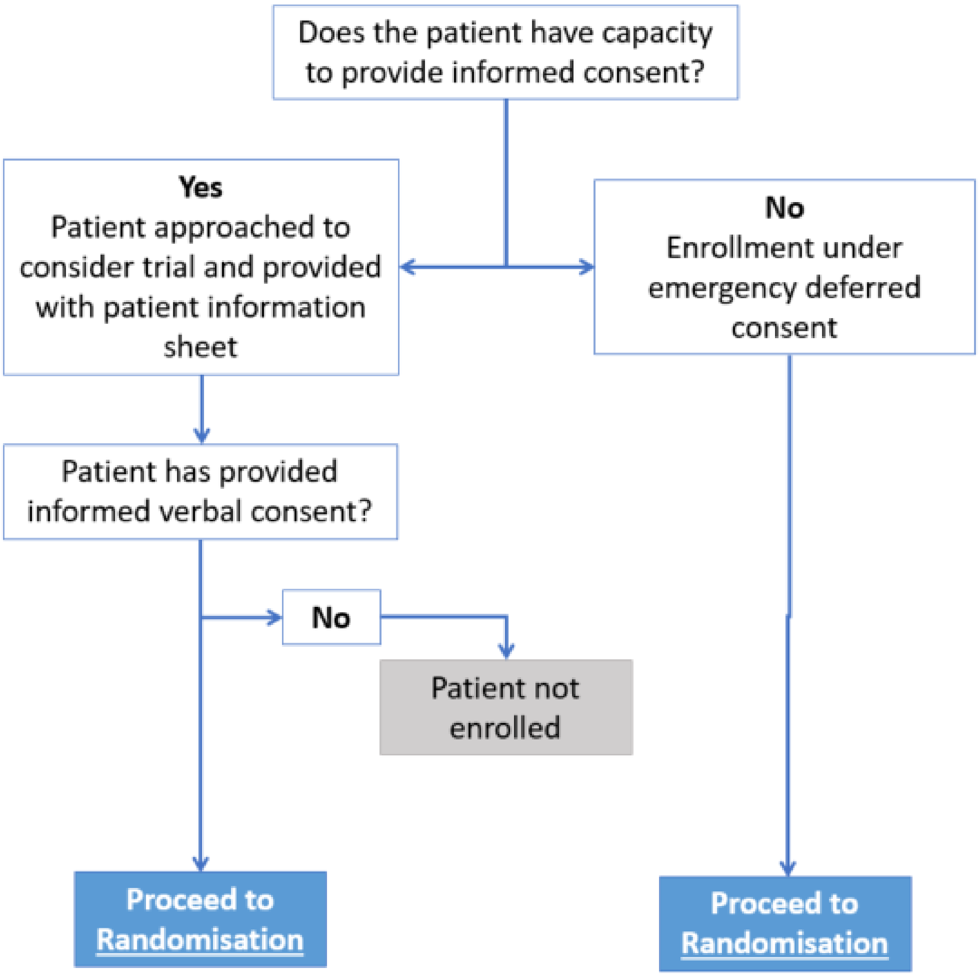

Scotland

Patients will be assessed for capacity. Patients with capacity will be provided with verbal information about the trial and given a copy of the written participant information sheet. They will be given adequate time to review the information and ask questions. Due to ongoing concerns regarding risk of infection transmission, verbal consent only will be sought and documented on a study form and in the patient hospital records. Where a patient declines participation at this point they will not be entered into the study.

For patients that lack capacity, it is not practical or appropriate to consult a guardian/welfare attorney prior to enrolment without placing the potential participant at risk of harm from delaying treatment. These processes will also not be feasible or safe to undertake in order to minimise risk of viral transmission to those involved. Significant limitations have been placed on the visitation of COVID-19 patients and therefore a guardian is unlikely to be immediately be available, and to attempt contact via telephone will delay treatment. Therefore these patients will not be entered into the study, see figure 3.

Figure 3. Consent processes for Scotland

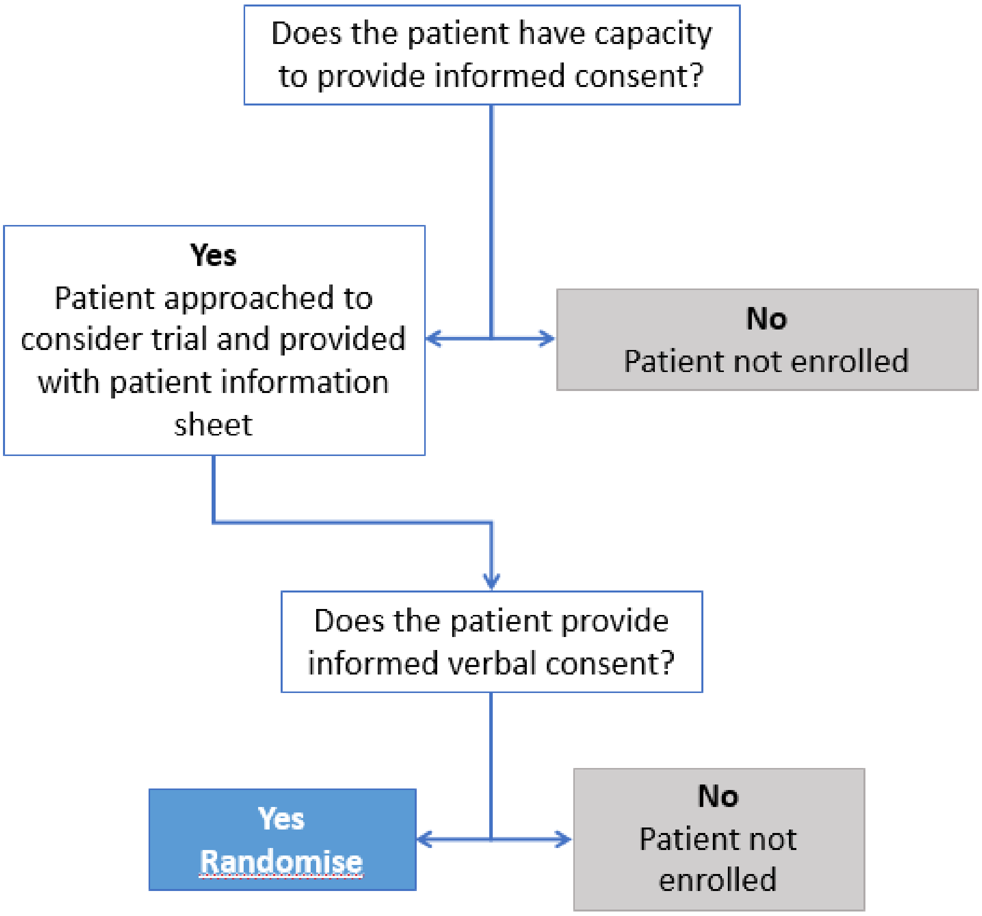

2.8.1 Consent following initial enrolment (for England, Wales and Northern Ireland only)

Following enrolment, the participant will be followed up regularly by the hospital research team. In participants that regain capacity, the site research team will approach the participant and provide verbal information about the trial. They will also be provided with the written participant information sheet, and given adequate time to review the information sheet and ask questions. Due to ongoing concerns regarding risk of infection transmission, verbal consent only will be sought and documented on a study form and in the patient hospital records.

In participants that do not regain capacity by hospital discharge, an approach will be made to a personal consultee or professional consultee. The initial approach should ideally be made to a personal consultee. However, this will likely be precluded due to visiting restrictions, in which case the opinion of a professional consultee will be sought. The professional consultee will be a registered medical practitioner that is independent of the trial. The consultee will be provided with a verbal explanation of the trial and a copy of the written participant information sheet and cover note. If the consultee does not know of any objection to research participation expressed by the participant, they will be asked to verbally confirm this. This will be recorded on a study record form. Procedures for consent following enrolment are different in the three jurisdictions (England & Wales, Scotland, Northern Ireland). Consent processes in Northern Ireland will follow the Exception for Urgent treatment in Part 8 of the Mental Capacity Act (Northern Ireland) 2016. For this trial, the consent processes used in England & Wales will be used in Northern Ireland, with the exception that Nominated Person assent will be taken and no Nominated Consultees will be appointed. These procedures will be updated in line with updated legislation.

Under the emergency regulations that have been introduced for COVID-19 related research, in the event that the participant dies prior to regaining capacity and before consent or consultee agreement can be sought, patient identifiable information may be retained by the WCTU to complete trial analysis as per the protocol and statistical analysis plan, for sites in England, Wales and Scotland. Patient identifiable information will not be transferred or retained by the WCTU for participants in Northern Ireland that do not provide consent.

On occasion, participant consent or personal/professional consultee agreement may not be obtained prior to hospital transfer or discharge. In this circumstance, the site research team will attempt to contact the participant or their consultee (if it is known that the participant lacks mental

capacity) at their place of residence to seek agreement. Up to three attempts to contact the participant will be made. Given the urgent need to answer this research question, for sites in England, Wales and Scotland, minimal personal identifiable data (NHS or CHI number, date of birth, postcode) will be submitted contemporaneously to Warwick Clinical Trials Unit. Patient identifiable information will not be transferred or retained by the WCTU for participants in Northern Ireland that do not provide consent. In the event that a trial participant declines consent to continue in the trial, personal identifiable data will be deleted. Information collected about the participant up to the point of withdrawal will be retained for analysis.

2.9 Randomisation

2.9.1 Randomisation

In this trial, there may well be the possibility that the devices will not be available as a result of the challenging environment the clinical staff are presented with. For this reason, we will set up two randomisation systems, which will be integrated to maintain balance.

Prior to randomisation, the clinician/nurse will check the availability of the devices. If no device is available then there will be no randomisation. If one of the devices is available (either CPAP or HFNO), then the clinician/nurse will choose the randomisation procedure which allocates standard care and CPAP or HFNO (system 1). If both devices are available, then system 2 will allow for an allocation using the three arms (standard care, CPAP or HFNO). These two systems will be integrated and constantly updated to ensure that the allocation ratio (1:1:1 for standard care: CPAP: HFNO) is maintained if possible. There is a possibility that this ratio will not be maintained and this has been compensated for in our sample size, which has been inflated accordingly.

A simple and secure, interactive voice response (IVR) randomisation system has been established by the programming team at Warwick Clinical Trials Unit. A computer-generated randomisation sequence has been generated by the minimisation method. Patients will be stratified by site, gender (M/F), and age (<50, >=50 years). If a patient has been randomised and found to be ineligible or the device is not available, they will be included in the analysis on an intention-to-treat basis. Any data already collected will be retained and included in the analysis unless otherwise indicated.

A back-up emergency randomisation system is currently being developed and full details will be made available and maintained on the trial website.

2.9.2 Post-randomisation withdrawals, exclusions and Moves out of Region

Participants may be discontinued from the trial treatment and/or the trial at any time without prejudice. Unless a participant explicitly withdraws their consent, they should be followed-up wherever possible and data collected as per the protocol until the end of the trial.

2.10 Trial treatments / intervention

Patients in all three treatment groups will be intubated and receive invasive mechanical ventilation if clinically required.

2.10.1 Continuous Positive Airway Pressure arm

Continuous positive airway pressure will be administered according to local protocol/guidelines. Details of initial settings (CPAP Pressure and FiO_2) will be recorded on the CRF. Administration of CPAP will not be protocolised and left to clinical discretion for management.

2.10.2 High Flow Nasal Oxygen

High flow nasal oxygen will be administered according to local protocol/guidelines. Details of initial settings (Flow and FiO_2) will be recorded on the CRF. Administration of HFNO will not be protocolised and left to clinical discretion for management.

2.10.3 Standard care

Standard care will comprise regular ward level oxygen therapy according to local protocol/guidelines, and excludes escalation to CPAP and HFNO. Administration of regular oxygen therapy will not be protocolised and left to clinical discretion for management.

2.11 Blinding and contamination

2.11.1 Methods for ensuring blinding

This is an open-label study. However, while the study is in progress, access to tabular results by treatment allocation will not be available to the research team, patients, or members of the Trial Steering Committee (unless the Data Safety Committee advises otherwise).

In addition, the clinical staff delivering the interventions will not be blinded or masked, and therefore there is possibility that bias could be introduced by knowledge of treatment allocation through administration of interventions. Due to the exceptional circumstance of this study, it is not possible to protocolise the interventions or provide training for clinical staff to ensure that delivery of the treatment is standardised across the trial sites. We will assess this bias in the interim stages of the trial and present the trial data by each site and intervention to the Data Monitoring

Committee and Trial Steering Committee. Both our primary and secondary outcomes are objective and this will ensure that detection bias is reduced.

2.11.2 Contamination

It is likely that the intervention the patient is randomised to may not be the one that is allocated or the patient may have crossed over to one of the other interventions. In this case, we will still include the patient in the trial on an intention-to-treat bias. However, we will do additional statistical analyses which will accommodate for patients who did not conform to the allocated intervention by means of a compliers analysis (as detailed in the statistical analysis section).

2.12 End of trial

The study will end when 4002 participants have been randomised and the last participant has completed final follow-up. The study will be stopped prematurely if:

- Mandated by the Ethics Committee
- Following recommendations from the Data Monitoring Committee (DMC)
- Funding for the trial ceases

The Ethics Committee that originally gave a favourable opinion of the study will be notified in writing if the study has been concluded or terminated early.

3 METHODS AND ASSESSMENTS

3.1 Schedule of delivery of intervention and data collection

Table 2. Trial assessments

**Visit**	**1**	**2**	**3**	**4**
**Visit Window**<br>(No. Weeks ± No. Days)	**Day 0**<br>Baseline	**Regular in-hospital reviews**<br>(at least weekly)	**Day 30**<br>(+/- 2 days)†	**Hospital discharge†**
Baseline data collection	✓			
Randomisation	✓			
Intervention	✓*	✓*		
Adverse events		✓	✓	✓
Survival status		✓	✓	✓
Outcome assessment			✓	✓

*- Intervention will be delivered up to point of death, tracheal intubation, or clinical determination that no ongoing need (palliation or improvement).

†- Participants to be followed-up to day 30 or hospital discharge, whichever comes later

4 ADVERSE EVENT MANAGEMENT

4.1 Definitions

4.1.1 Adverse Events (AE)

An Adverse Event (AE) is defined as any untoward medical occurrence in a trial participant and which does not necessarily have a causal relationship with the treatment/intervention. Only AEs clearly related to the use of CPAP or HFNO or expected will be recorded.

The following are expected adverse events and will be recorded in the CRF; they predominantly relate to the use of CPAP, as there are minimal AEs reported for HFNO:

- Interface intolerance due to excessive air leaks
- Pain
- Cutaneous pressure sore or pressure area
- Claustrophobia
- Oro-nasal dryness
- Respiratory acidosis with pH <7.25 prior to intubation
- Haemodynamic instability
- Vomiting
- Aspiration of gastric contents

4.1.2 Serious Adverse Events (SAEs)

A Serious Adverse Event (SAE) is an AE that fulfils one or more of the following criteria:

- Results in death
- Is immediately life-threatening
- Requires hospitalisation or prolongation of existing hospitalisation
- Results in persistent or significant disability or incapacity
- Is a congenital abnormality or birth defect
- Immediate intervention was required to prevent one of the above or is an important medical condition.

4.1.3 Assessing and Reporting AEs, SAEs and related SAEs

Adverse events

These events will be included as part of the safety analysis for the trial and do not need to be reported separately to the Trial Coordinating Centre. The local PI will be responsible for confirming the relatedness of any AE to the trial intervention. If an AE occurs, the clinician responsible for the patient should decide whether it is safe to continue CPAP, with or without modification, or whether CPAP should be discontinued. AEs will be reviewed/monitored by the TMG for trends.

Serious adverse events

Serious adverse events (SAE), occurring from the time of randomisation until 30 days post cessation of the trial intervention must be recorded on the Serious Adverse Event reporting form in the participant's CRF and emailed to WCTU (WCTUQA@warwick.ac.uk) within 24 hours of the research staff becoming aware of them. Events that do not need reporting as SAEs are listed below.

For each SAE the following information will be collected from the investigator site:

- full details in medical terms and case description
- event duration (start and end dates, if applicable)
- action taken
- outcome
- seriousness criteria
- causality (i.e. relatedness to intervention), in the opinion of the investigator (Table 3)

Once received, an independent causality assessment will be undertaken by the CI or clinical delegate. For any SAEs which are suspected to be caused by the trial intervention by either the CI or site clinician, expectedness will be confirmed by the CI or a delegate at WCTU. SAEs that are deemed to be unexpected and related to the study will be notified to the Research Ethics Committee (REC) within 15 days. All such events will be reported to the Sponsor, Trial Steering Committee and Data Monitoring Committee at their next meetings. All participants experiencing SAEs will be followed-up until the event has been resolved or a final outcome has been reached as per protocol until the end of the trial.

Table 3. Relationship of SAEs to trial intervention

Relationship to trial intervention	Description
Unrelated	There is no evidence of any causal relationship
Unlikely to be related	There is little evidence to suggest there is a causal relationship (e.g.

	the event did not occur within a reasonable time after administration of the trial intervention or device). There is another reasonable explanation for the event (e.g. the patient's clinical condition, other concomitant treatment).
Possible relationship	There is some evidence to suggest a causal relationship (e.g. because the event occurs within a reasonable time after administration of the trial intervention or device). However, the influence of other factors may have contributed to the event (e.g. the patient's clinical condition, other concomitant treatments). This event will be reported as related.
Probable relationship	There is evidence to suggest a causal relationship and the influence of other factors is unlikely. This event will be reported as related.
Definitely related	There is clear evidence to suggest a causal relationship and other possible contributing factors can be ruled out. This event will be reported as related.

4.1.4 SAEs Exempt from reporting

Because the trial is recruiting a population that is already in a life-threatening situation, it is expected that many of the participants will experience SAEs. Events that commonly occur in this population and those that are collected as outcomes of the trial should not be reported as SAEs. Common events, not for reporting include:

- Death
- Organ failure
- Pneumonia
- Intubation
- Tracheostomy

4.2 Responsibilities

<u>Principal Investigator (PI)</u>:

Checking for AEs

1. Using medical judgement in assigning seriousness and causality
2. Ensuring that all SAEs are recorded and reported to WCTU (to receive on behalf of the Sponsor) within 24 hours of becoming aware of the event and provide further follow-up information as soon as available. Ensuring that SAEs are chased with WCTU if a record of receipt is not received within 2 working days of initial reporting.

3. Ensuring that AEs are recorded and reported to WCTU (to receive on behalf of the Sponsor) in line with the requirements of the protocol.

Chief Investigator (CI)/delegate or independent clinical reviewer:

1. Clinical oversight of the safety of patients participating in the trial, including an ongoing review of the risk / benefit.
2. Using medical judgement in assigning causality review of any specific SAEs in accordance with the trial risk assessment and protocol as detailed in the Trial Monitoring Plan.
3. Production and submission of annual reports to the REC.

Sponsor or delegate:

1. Central data collection and verification of AEs and SAEs, according to the trial protocol.
2. Expectedness assessment of related SAEs.
3. Reporting safety information to the CI, delegate or independent clinical reviewer for the ongoing assessment of the risk/benefit according to the Trial Monitoring Plan.
4. Reporting safety information to the independent oversight committees identified for the trial (Data Monitoring Committee (DMC) and/or Trial Steering Committee (TSC)) according to the Trial Monitoring Plan.
5. Expedited reporting of related and unexpected SAEs to the REC within required timelines.
6. Notifying Investigators of related and unexpected SAEs that occur within the trial.

Trial Steering Committee (TSC):

In accordance with the Trial Terms of Reference for the TSC, periodically reviewing safety data and liaising with the DMC regarding safety issues.

Data Monitoring Committee (DMC):

In accordance with the Trial Terms of Reference for the DMC, periodically reviewing unblinded overall safety data to determine patterns and trends of events, or to identify safety issues, which would not be apparent on an individual case basis.

4.3 Notification of deaths

Death is collected as a study outcome. No separate notification of death is required.

4.4 Reporting urgent safety measures

If any urgent safety measures are taken the CI/Sponsor shall immediately, and in any event no later than 3 days from the date the measures are taken, give written notice to the relevant REC of the measures taken and the circumstances giving rise to those measures.

5 DATA MANAGEMENT

5.1 Data collection and management

We will use the standard CTU study web-based application for study management and to record participant data (including case report forms) in accordance with the protocol. Clinical data will be collected during the ICU stay up to 30 days after randomisation or hospital discharge, whichever is later. Baseline characteristics collected include patient demographics, comorbidities, pre-admission function, inclusion/exclusion criteria, consent, time and date of randomisation, temperature at hospital admission, and COVID-19 diagnosis status. Data captured following randomisation will include initial device settings, duration of ventilation, prone therapy, ICU/hospital admission status, patient observations, AEs (treatment failure, need for other treatments, renal function, renal replacement therapy), survival status. Confirmation of COVID-19 diagnosis at any point will be recorded. Ethnicity, socioeconomic status and mortality will be reported from hospital records up until discharge and tracked after discharge using NHS Digital, the ICNARC and Research Centre and equivalent NHS data linkage in the devolved nations. ICU and hospital length of stay will be obtained from local centres or data linkage if appropriate.

The case report form (CRF) has been developed by the CTU and made available to the participating sites as a paper and electronic CRF (eCRF) for ease of data collection; supporting materials will be available to staff. All documents will be stored safely in confidential conditions. On all trial-specific documents, other than the signed consent, the participant will be referred to by a unique trial-specific number/patient identifier/code in any database, not by name. The trial will be conducted in accordance with the current approved protocol, Good Clinical Practice (GCP), relevant data protection regulations and standard operating procedures (SOPs). A monitoring plan and risk assessment will be devised to protect participant safety and integrity of trial data.

5.2 Database

The database has been developed by the Programming Team at WCTU and all specifications (i.e. database variables, validation checks, screens) agreed between the programmer and appropriate trial staff.

5.3 Data storage

All essential documentation and trial records will be stored by WCTU in conformance with the applicable regulatory requirements and access to stored information will be restricted to authorised personnel.

5.4 Data access and quality assurance

All data access will be controlled by generic usernames and passwords, and any changes to data will require the user to enter their username and password as an electronic signature in accordance with regulatory requirements. Staff will have access restricted to the functionality and data that are appropriate for their role in the study.

5.5 Archiving

Trial documentation and data will be archived for at least ten years after completion of the trial. Trial Master File and associated data will be archived by WCTU; trial data generated at study sites will be archived according to local policy.

6 STATISTICAL ANALYSIS

6.1 Power and sample size

The maximum total sample size will be 4002 patients over the three intervention arms (1334 per arm).

The primary end point will be a binary composite outcome of intubation or mortality within 30 days. An event (1) will be defined as intubation or death and an event (0) will be defined as no requirement for intubation and alive.

In a systematic review and meta-analysis to evaluate the evidence for the use of HFNO against usual care in ARF[8], the rate of mortality was 8.1% (90/1106) on the usual care arm and the rate of intubation was 15.7% (204/1300). A large UK based multi-centre open-labelled randomised control trial (3CPO) of 1069 patients presented with severe acute cardiogenic pulmonary oedema at 26 emergency department, compared standard oxygen therapy, CPAP and NIV, found a 7 day mortality rate of 9.8% and a mean intubation rate of 2.9% on the standard care arm. This is similar to the intubation rates of 2.7% reported in the British Thoracic Society's *National Respiratory Audit Programme Annual Report 2011/12*[22]. In a large systematic review and cost-effectiveness evaluation[23] of pre-hospital NIV for ARF, the intubation rate on usual rate ranged from 4.6%-17.7% and mortality at 30 days was found to be 9.3%-12.8%. In relation to COVID-19 infection, a very

recent publication in the Lancet using WHO data on the cumulative number of deaths to March 1, 2020, mortality rates were reported to be 5·6% (95% CI 5·4–5·8) for China and 15·2% (12·5–17·9) outside of China[1]. Furthermore, in another study relating to the COVID-19, which assessed, lopinavir- ritonovir and standard care, in hospitalised adults with confirmed SARS-Cov-2 infection and with an oxygen saturation of <=94%, the mortality rate on standard care at day 28 was 25% [24].

In our sample size calculation, we have assessed both outcome measures and taken the largest in terms of the incidence, to ensure we have maximum power and sample size for our study. Therefore taking the data from Baud *et al*[1] to be representative of usual care and assuming a conservative incidence of 15% for the composite outcome of intubation or mortality on the usual care arm, then using a two-sided 5% significance level, a total 3000 patients (1000 per arm across 3 arms) would give approximately 90% power to detect an odds ratio of 0.625, corresponding to a reduction from 15% to 10% in the composite event rate. allowing for the 11 interim analyses. Our comparisons will be based on CPAP versus standard care and HFNO versus standard care and allow for the formal interim analyses as agreed with the DMC.

However, there remain many uncertainties regards the sample size parameters and also whether an equal randomisation allocation ratio will be maintained. For this reason we have inflated our sample size to 4000 patients (4002 across the three arms) to maintain the study power and detect small effect sizes, if they exist. It may allow us to also assess the difference between CPAP and HFNO, if difference between these interventions are small enough to be detected with this larger sample size. Furthermore, it may be likely that we will not need all these patients if the trial stops at a formal interim analysis or if the DMC recommends that an arm is dropped.

The formal stopping rules will be set out in the statistical analysis plan, following discussions with the DMC. These criteria will be used to accept or drop an intervention arm, based on the assessment of the primary outcome at each interim point.

6.2 Statistical analysis of efficacy and harms

6.2.4 Statistics and data analysis

The statistical analysis will be based on intention to treat and will be conducted in line with the CONSORT statement[25]. For the overall analyses, numbers of events will be assessed using the logistic regression unadjusted and adjusted for important predictors, such as previous morbidities, age and gender) and results will be presented using odds ratios (with 95% confidence intervals). At the interim stages, the numbers of events will be analysed using the latter methods and compared

to the p-value critical values. A final p-value and treatment effect estimate will be calculated adjusting for the interim analyses using the methods described by Jennison and Turnbull[26], Fairbanks and Madsen[27] and, Emmerson and Fleming[28]. Intubation rates and mortality at 30 days will be assessed using the logistic regression models (both for adjusted and unadjusted analyses) and summarised using odds ratio (with 95% confidence intervals). Continuous secondary data will be presented using summary statistics and analysed using parametric or non-parametric methods depending on the distribution of the data.

If possible, we will conduct a compliers average cause effect (CACE) analysis to assess the effect on the non-compliance to the allocation interventions (for all three interventions or two, if one is dropped)[29].

We anticipate no or very little missing data. However, in the case of missingness, we will use multiple imputation techniques (ICE procedures) to assess the missingness mechanisms.

6.2.5 Planned recruitment rate

Recruitment will take place in 40 adult ICUs and centres to treat COVID-19 patients across the UK (England, Wales, Scotland and Northern Ireland) with a track record of delivery on critical care research, in order to facilitate enrolment of the required number of patients and ensure relevance to the wider NHS. Assuming a maximum sample size of 4002 patients, each site would enrol approximately 100 patients over the planned 12 months duration for recruitment.

Due to the rapidly evolving clinical landscape in the context of COVID-19, estimating a monthly recruitment rate is challenging; we anticipate the majority of patients would be recruited in the first 6 months but there is the possibility that seasonal fluctuations could result in further presentations in the winter months. We have therefore elected to focus on an overall recruitment number per site to accommodate any variations in monthly recruitment.

In 2018-19, 83% of ICUs in England actively recruited to the NIHR Portfolio of critical care research studies. In addition, the Chief Investigators have extensive experience of leading and participating in successful large-scale, UK multi-centre trials, including the devolved nations. We will employ the NIHR Critical Care National Specialty Group database, the UK Critical Care Research Group membership, and sites previously involved in critical care research studies, to identify sites.

6.3 Health Economic Evaluation

There will be no health economic evaluation within this trial.

7 TRIAL ORGANISATION AND OVERSIGHT

7.1 Sponsor and governance arrangements

The University of Warwick will sponsor the study.

Sub-contracts delegating responsibilities to research sites will be established using our standard contracting processes with NHS organisations.

7.2 Ethical approval

All required ethical approval(s) for the trial will be sought using the Integrated Research Application System. The trial will be conducted in accordance with all relevant regulations. Before enrolling patients into the trial, each trial site must ensure that the local conduct of the trial has agreement of the relevant NHS Trust Research & Development (R&D) department. Sites will not be permitted to enrol patients into the trial until all required agreements are in place.

Annual reports will be submitted to the REC within 30 days of the anniversary date on which the favourable opinion was given, and annually until the trial is declared ended. The REC will be notified of the end of the trial (whether at planned time or prematurely). The CI will submit a final report to the required authorities with the results, including any publications within one year of the end of the trial.

Peer review for this protocol will initially be provided by the Department of Health and Social Care and the NIHR Urgent Public Health prioritisation group. Additional peer review will be obtained as required.

7.3 Trial Registration

The trial is registered in advance of recruitment commencing, on the ISRCTN database (https://www.isrctn.com/).

Registration confirmed: ISRCTN16912075

7.4 Notification of serious breaches to GCP and/or trial protocol

Trial protocol deviation and violations

Deviations from clinical trial protocols and GCP occur commonly in clinical studies. The majority of these instances are technical deviations that do not result in harm to the trial subjects or significantly affect the scientific value of the reported results of the trial. Violation is a failure to

comply with or variance from GCP and/or the final approved protocol. This results from error, fraud or misconduct. These cases should be documented in the protocol deviation and violation section of the case report form for the trial and appropriate corrective and preventative actions taken. Deviations will be included and considered when the clinical study report is produced, as they may have an impact on the analysis of the data.

Serious breach

A “serious breach” is a breach which is likely to effect to a significant degree –

(a) the safety or physical or mental integrity of the subjects of the trial; or
(b) the scientific value of the trial

The sponsor will be notified immediately of any case where the above definition applies during the trial conduct phase, and will notify the licensing authority in writing of any serious breach of

(a) the conditions and principles of GCP in connection with that trial; or
(b) the protocol relating to that trial, as amended from time to time, within 7 days of becoming aware of that breach

7.5 Indemnity

NHS indemnity covers NHS staff, medical academic staff with honorary contracts, and those conducting the trial. NHS bodies carry this risk themselves or spread it through the Clinical Negligence Scheme for Trusts, which provides unlimited cover for this risk. The University of Warwick provides indemnity for any harm caused to participants by the design of the research protocol.

7.6 Trial timetable and milestones

These milestones are estimated and subject to change given the rapidly evolving circumstances under which this trial will be conducted.

	Month	Recruitment
Set-up	1	n/a
Recruitment	2-13	4002
Follow up	14	n/a
Analysis	15-18	n/a

7.7 Administration

Trial coordination will be based at WMS/WCTU, University of Warwick. All day-to-day coordination of the study will be the responsibility of the Trial Manager. All clinical coordination of the study will be the responsibility of Professor Gavin Perkins. The study is managed by a multi-disciplinary team.

The study office team will assist and facilitate the setting up of centres wishing to collaborate in the study. In addition the study office team will:

- Distribute the standardised data collection forms to collaborators
- Organise the telephone randomisation service for formal study entry
- Monitor the collection of data, process data and seek missing data
- Train local staff with regards to data collection remotely
- Ensure the confidentiality and security of all study forms and data
- Conduct extensive data checking and cleaning
- Organise any interim and main analyses
- Organise Steering Committee, DMC and Collaborators meetings

The study office will receive completed data forms, via the online web application or email. Upon receipt, data forms will be checked for completeness and entered into a study specific dedicated computer programme which will check the data validity.

7.8 Trial Management Group (TMG)

The Trial Management Group, consisting of the project staff and co-investigators involved in the day-to-day running of the trial, will meet regularly throughout the project. Significant issues arising from management meetings will be referred to the Trial Steering Committee or Investigators, as appropriate.

7.9 Trial Steering Committee (TSC)

The trial will be guided by a group of respected and experienced personnel and trialists as well as at least one 'lay' representative. The TSC will have an independent Chairperson. Meetings will be held via teleconference or video conference at regular intervals determined by need but not less than once a year. Routine business is conducted by email, post or teleconferencing.

The Steering Committee, in the development of this protocol and throughout the trial will take responsibility for:

- Major decisions such as a need to change the protocol for any reason

- Monitoring and supervising the progress of the trial
- Reviewing relevant information from other sources
- Considering recommendations from the DMC
- Informing and advising on all aspects of the trial

Membership of the TSC it to be confirmed. The full remit and responsibilities of the TSC will be documented in the Committee Charter which will be signed by all members.

7.10 Data Monitoring Committee (DMC)

A DMC will be appointed comprising of two independent clinicians with experience in clinical trials and an independent statistician. They will be provided with interim reports with outcome measures, when data are available on 1000 patients, at 4 time points in the study. One of the independent clinicians will have experience in undertaking clinical trials in emergency or acute care. The DMC charter will be based on the DAMOCLES study group template.[35] Its roles will include: monitoring the data and making recommendations to the TSC on whether there are any ethical or safety reasons why the trial should not continue; making recommendations as to whether to drop any arm from the study for futility, either relative to standard care or to other arms; advising the TSC regarding the release of data and/or information; considering data emerging from other related studies.

Membership of the DMC will be confirmed. DMC meetings will also be attended by the Chief Investigator and Trial Manager (for non-confidential parts of the meeting) and the trial statistician. The full remit and responsibilities of the DMC will be documented in the Committee Charter which will be signed by all members.

7.11 Essential Documentation

A Trial Master File will be set up according to Warwick University SOP and held securely at the coordinating centre. The coordinating centre will provide Investigator Site Files to all recruiting centres involved in the trial.

7.12 Financial Support

A full breakdown of costs, specifically ‘Research’ and ‘NHS Service Support’ costs, will be provided in due course. The study will be included on the NIHR Portfolio and is eligible for NHS Service Support costs.

8 MONITORING, AUDIT AND INSPECTION

Local monitoring of protocol compliance

In this pragmatic trial, the precise delivery of study interventions will be at the discretion of the treating clinical team. We will record the delivery of interventions on the case report form, including unanticipated crossover between study arms.

Monitoring

On-site monitoring will not be feasible in this trial, due to restrictions on non-essential staff visiting hospitals with COVID-19 patients. A risk-based proportionate approach outlined in the monitoring plan for off-site monitoring will be developed through discussion with the trial sponsor, that takes account of the challenging circumstances in which this trial will operate and the extreme pressure that will be placed on hospital staff.

Reporting

Protocol deviations or violations (and actions taken to prevent recurrence) will be recorded in the case report form. Serious breaches of the study protocol or GCP should be immediately reported to the Chief Investigator. The Chief Investigator in consultation with the PI will take whatever immediate action is required to safeguard the wellbeing of participant. The Chief Investigator will notify the Sponsor immediately and Ethics committee within 7 days of becoming aware of the serious breach.

9 CO-ENROLMENT

Co-enrolment with other trials will be reviewed on a case-by-case basis in accordance with national NIHR-supported co-enrolment guidelines[30]. There are many current examples of successful co-enrolment between UK critical care studies, facilitated by these guidelines.

10 PATIENT AND PUBLIC INVOLVEMENT (PPI)

Three members of Royal College of Anaesthetist Patient Carer Public Involvement Engagement (PCPIE) were approached to give their opinion on research question, study design, appropriateness of emergency consent and patient facing documents. PCPIE members were unanimous in their support for the research question and considered the study to be highly relevant to patients and public. They opined that it is critical and urgent to find the optimal breathing support for patients to avoid invasive ventilation and improve patient survival. They were also particularly supportive of the adaptive pragmatic study design and planned rapid dissemination of study results. Emergency

consent was considered both appropriate in a very difficult and time critical situation and sensitive in limiting burden to patient and family.

Furthermore, we also consulted with an independent member of the Clinical Research Ambassador Group, University Hospitals Birmingham NHS Foundation Trust with extensive experience of contributing to emergency care trials. This PPI colleague was supportive and confirmed our approach of emergency consent given the challenging clinical circumstances and urgency with which treatment of potential benefit should be delivered to patients, advising that they felt it would be considered acceptable to patients and members of the public. Planned use of data without consent was also agreed as being necessary, and acceptable.

In addition, investigating optimum ventilation practice was prioritised in a 2014 James Lind Alliance Priority Setting Partnership for intensive care research involving patients and family representatives[31], and subsequently in a NIHR critical care research priority setting survey[32]. We will continue to engage patients and family members at later stages of the trial conduct where possible, such as dissemination of findings.

11 CONFIDENTIALITY

The University of Warwick (Research Impact Services, University of Warwick, Coventry CV4 7AL) is the Sponsor for the study. The study is being conducted in full adherence with the principles of the Declaration of Helsinki and MRC Good Clinical Practice principles and guidelines. It also complies with all applicable UK legislation and Warwick Standard Operating Procedures. All data are being stored securely and held in accordance with the Data Protection Act 2018. All identifiable data are pseudonymised and treated as confidential. All CRFs, questionnaires, study reports and communication regarding the study will identify the patients by the assigned unique trial identifier and initials only. Patient confidentiality will be maintained at every stage and identifiable information will not be made publicly available to the extent permitted by the applicable laws and regulations. The trial consent process ensures that patients have the choice of whether or not to continue to participate in data collection and are given all relevant information about the study to make an informed decision. Participants are informed that they are free to withdraw from the trial at any time during any phase without providing a reason and without prejudice, if they so wish.

12 DISSEMINATION AND PUBLICATION

The approach will be informed by WCTU SOP 22 ‘Publication & Dissemination’.

The results of the trial will be reported first to trial collaborators. The main report will be drafted by the trial coordinating team, and the final version will be agreed by the Trial Steering Committee before submission for publication, on behalf of the collaboration. The success of the trial depends on the collaboration of doctors, nurses and researchers from across the UK. Equal credit will be given to those who have wholeheartedly collaborated in the trial.

The trial will be reported in accordance with the Consolidated Standards of Reporting Trials (CONSORT) guidelines (www.consort-statement.org).

The results of the trial will be shared widely, and participants are able to request a copy of the results through contacting the local study team. Following the conclusion of the trial, summary information will be sent to surviving participants who recorded a desire to receive this information.

13 RESILIENCE

Given the circumstances under which this trial will be conducted, we have specifically built in resilience to support all relevant personnel involved. There are two Chief Investigators, two statisticians, five further respiratory/critical care physicians, two respiratory physiotherapists, and key WCTU representation, with additional access to the full team of trial managers, coordinators and other staff within the CTU. In addition, we adopt the same approach when identifying members of the TSC and DMC.

14 REFERENCES

1. Baud D, Qi X, Nielsen-Saines K, et al. Real estimates of mortality following COVID-19 infection. *The Lancet Infectious Diseases* 2020;Published Ahead of Print doi: 10.1016/S1473-3099(20)30195-X
2. The Centre of Evidence based Medicine (CEBM) – University of Oxford. England and Wales Mortality during the COVID-19 outbreak. https://www.cebm.net/covid-19/england-and-wales-mortality-during-the-covid-19-outbreak/.
3. ICNARC report on COVID-19 in critical care (27 March 2020). 2020;Intensive Care National Audit and Research Centre, London, UK
4. Guan W-j, Ni Z-y, Hu Y, et al. Clinical Characteristics of Coronavirus Disease 2019 in China. *N Engl J Med* 2020;Published Ahead of Print doi: 10.1056/NEJMoa2002032
5. Wu Z, McGoogan JM. Characteristics of and Important Lessons From the Coronavirus Disease 2019 (COVID-19) Outbreak in China: Summary of a Report of 72 314 Cases From the Chinese Center for Disease Control and Prevention. *JAMA* 2020;Published Ahead of Print doi: 10.1001/jama.2020.2648
6. Alhazzani W, Hylander Møller M, Arabi Y, et al. Surviving Sepsis Campaign: Guidelines on the Management of Critically Ill Adults with Coronavirus Disease 2019 (COVID-19). *Intensive Care Med* 2020;Published Ahead of Print doi: DOI: 10.1007/s00134-020-06022-5
7. Corley A, Rickard CM, Aitken LM, et al. High-flow nasal cannulae for respiratory support in adult intensive care patients. *Cochrane Database of Systematic Reviews* 2017(5) doi: 10.1002/14651858.CD010172.pub2
8. Monro-Somerville T, Sim M, Ruddy J, et al. The Effect of High-Flow Nasal Cannula Oxygen Therapy on Mortality and Intubation Rate in Acute Respiratory Failure: A Systematic Review and Meta-Analysis. *Read Online: Critical Care Medicine | Society of Critical Care Medicine* 2017;45(4):e449-e56. doi: 10.1097/ccm.0000000000002091
9. Ni Y-N, Luo JL, Yu H, et al. The effect of high-flow nasal cannula in reducing the mortality and the rate of endotracheal intubation when used before mechanical ventilation compared with conventional oxygen therapy and noninvasive positive pressure ventilation. A systematic review and meta-analysis. *Am J Emerg Med* 2017;36(2):226-33.
10. Ou X, Hua Y, Liu J, et al. Effect of high-flow nasal cannula oxygen therapy in adults with acute hypoxemic respiratory failure: a meta-analysis of randomized controlled trials. *Can Med Assoc J* 2017;189(7):E260-E67. doi: 10.1503/cmaj.160570

11. Rochwerg B, Granton D, Wang DX, et al. High flow nasal cannula compared with conventional oxygen therapy for acute hypoxemic respiratory failure: a systematic review and meta-analysis. *Intensive Care Med* 2019;45(5):563-72. doi: 10.1007/s00134-019-05590-5
12. Frat J-P, Thille AW, Mercat A, et al. High-Flow Oxygen through Nasal Cannula in Acute Hypoxemic Respiratory Failure. *N Engl J Med* 2015;372(23):2185-96. doi: 10.1056/NEJMoa1503326
13. The Australian and New Zealand Intensive Care Society (ANZICS) COVID-19 Guidelines, Version 1. 2020;ANZICS; Available at www.anzics.com.au
14. Faria DAS, da Silva EMK, Atallah Á, et al. Noninvasive positive pressure ventilation for acute respiratory failure following upper abdominal surgery. *Cochrane Database of Systematic Reviews* 2015(10) doi: 10.1002/14651858.CD009134.pub2
15. Bakke SA, Botker MT, Riddervold IS, et al. Continuous positive airway pressure and noninvasive ventilation in prehospital treatment of patients with acute respiratory failure: a systematic review of controlled studies. *Scandinavian Journal of Trauma, Resuscitation and Emergency Medicine* 2014;22(1):69. doi: 10.1186/s13049-014-0069-8
16. Williams TA, Finn J, Perkins GD, et al. Prehospital Continuous Positive Airway Pressure for Acute Respiratory Failure: A Systematic Review and Meta-Analysis. *Prehosp Emerg Care* 2013;17(2):261-73. doi: 10.3109/10903127.2012.749967
17. Guidance for the role and use of non-invasive respiratory support in adult patients with coronavirus (confirmed or suspected); 26 March 2020 Version 2. 2020;NHS England and NHS Improvement. Available at https://www.england.nhs.uk/coronavirus/wp-content/uploads/sites/52/2020/03/CLEARED_Specialty-guide_-NIV-respiratory-support-and-coronavirus-v2-26-March-003.pdf
18. Brochard L, Mancebo J, Wysocki M, et al. Noninvasive Ventilation for Acute Exacerbations of Chronic Obstructive Pulmonary Disease. *N Engl J Med* 1995;333(13):817-22. doi: 10.1056/nejm199509283331301
19. Rochwerg B, Brochard L, Elliott MW, et al. Official ERS/ATS clinical practice guidelines: noninvasive ventilation for acute respiratory failure. *Eur Respir J* 2017;50(2):1602426. doi: 10.1183/13993003.02426-2016
20. Yang X, Yu Y, Xu J, et al. Clinical course and outcomes of critically ill patients with SARS-CoV-2 pneumonia in Wuhan, China: a single-centered, retrospective, observational study. *Lancet Respiratory Medicine* 2020;Published Ahead of Print doi: 10.1016/S2213-2600(20)30079-5
21. Davies H, Shakur H, Padkin A, et al. Guide to the design and review of emergency research when it is proposed that consent and consultation be waived. *Emergency Medicine Journal* 2014;31(10):794-95. doi: 10.1136/emermed-2014-203675

22. British Thoracic Society. National Respiratory Audit Programme annual report. 2012. Available at: https://www.brit-thoracic.org.uk/document-library/audit-and-quality-improvement/audit-reports/bts-national-respiratory-audit-programme-annual-report-2011-2012/. Accessed May 1, 2013.
23. Pandor A, Thokala P, Goodacre S, et al. Pre-hospital non-invasive ventilation for acute respiratory failure: a systematic review and cost-effectiveness evaluation. *Health Technol Assess* 2015;19(42) doi: 10.3310/hta19420
24. Cao B, Wang Y, Wen D, et al. A Trial of Lopinavir–Ritonavir in Adults Hospitalized with Severe Covid-19. *N Engl J Med* 2020;Published Ahead of Print doi: 10.1056/NEJMoa2001282
25. Moher D, Hopewell S, Schulz KF, et al. CONSORT 2010 Explanation and Elaboration: updated guidelines for reporting parallel group randomised trials. *Br Med J* 2010;340:c332 doi: 10.1136/bmj.c869
26. Jennison C, Turnbull B. Group Sequential Methods with Applications to Clinical Trials. *Boca Raton: Chapman & Hall* 2000
27. Fairbanks K, Madsen R. P values for tests using a repeated significance test design. *Biometrika* 1982;69:69-74.
28. Emerson S, Fleming T. Parameter estimation following group sequential hypothesis testing. *Biometrika* 1990;77:875-92.
29. Long Q, Little RJA, Lin X. Estimating causal effects in trials involving multitreatment arms subject to non-compliance: a Bayesian framework. *Journal of the Royal Statistical Society: Series C (Applied Statistics)* 2010;59(3):513-31. doi: 10.1111/j.1467-9876.2009.00709.x
30. Krige A, Pattison N, Booth M, et al. Co-Enrolment to Intensive Care Studies – A UK Perspective. *Journal of the Intensive Care Society* 2013;14(2):103-06. doi: 10.1177/175114371301400203
